# OGI-RT-DETR: A Lightweight Vision-Sensing Model for Real-Time Graphite Ore Grade Recognition in Intelligent Sorting

**DOI:** 10.3390/s26144341

**Published:** 2026-07-08

**Authors:** Zhaojie Sun, Xueyu Huang, Dehui Fu, Guang Yang, Yipeng Li

**Affiliations:** 1 School of Industrial Software, Jiangxi University of Science and Technology, Nanchang 330013, China; 15070829913@163.com (Z.S.); fudehui963@gmail.com (D.F.); heliop710823@gmail.com (G.Y.); lvanio@163.com (Y.L.); 2 School of Electronic Information Industry, Jiangxi University of Science and Technology, Ganzhou 341600, China

**Keywords:** graphite ore grade recognition, intelligent sorting, vision-based sensing, edge devices, OGI-RT-DETR, feature fusion, multi-scale semantic interaction

## Abstract

Vision-based sensing is a practical approach for real-time ore grade recognition in intelligent mineral sorting. However, graphite ore images captured in industrial environments are often affected by illumination variation, background interference, surface oxidation, and subtle visual differences among grades, limiting the reliability of existing models on edge devices. To address these issues, this study proposes OGI-RT-DETR, a lightweight real-time end-to-end vision-sensing model for graphite ore grade recognition. Based on RT-DETR-r18, a PConv-Rep backbone enhancement module is designed to reduce redundant computation and improve the extraction of ore texture, edge, and irregular shape features. A CFPT feature fusion module is constructed to strengthen multi-scale semantic interaction and spatial fusion under complex backgrounds. A Wise-Focaler-MPDIoU bounding box regression loss is further proposed to improve localization stability and regression accuracy. Experiments were conducted on a self-built augmented graphite ore image dataset collected using an industrial camera system. The proposed OGI-RT-DETR achieved a Precision of 85.6%, Recall of 87.6%, mAP50 of 86.1%, and inference speed of 84.5 FPS. Compared with the baseline RT-DETR-r18, OGI-RT-DETR improved mAP50 by 3.7 percentage points and FPS by approximately 8.2%, while reducing FLOPs and parameters by 31.8% and 38.2%, respectively. These results indicate that OGI-RT-DETR can support accurate, efficient, and lightweight visual sensing for real-time graphite ore sorting.

## 1. Introduction

Graphite is a non-metallic mineral resource of significant strategic importance. Owing to its excellent electrical conductivity, thermal conductivity, high-temperature resistance, lubricity, and chemical stability, graphite has been widely used in metallurgy, refractory materials, machinery manufacturing, electronic information, aerospace, new energy batteries, and energy storage equipment [1,2]. In recent years, with the rapid development of new energy vehicles, lithium-ion batteries, and large-scale energy storage industries, the demand for high-quality graphite materials has continued to increase. In particular, during the preparation of anode materials for power batteries, the quality of graphite feedstock directly affects battery cycle life, energy density, and safety performance. Therefore, the efficient exploitation, precise sorting, and stable supply of graphite resources are not only closely related to the utilization efficiency of mineral resources, but also of great significance to the security of the new energy industrial chain and the development of green and low-carbon technologies [3,4].

Graphite ore grade is commonly used to characterize the content of fixed carbon or valuable carbonaceous components in ore, and serves as an important basis for evaluating the economic value of graphite resources, determining beneficiation processes, and guiding production scheduling [5]. In practical mining and processing operations, graphite ores of different grades may exhibit certain differences in color intensity, surface texture, reflectance characteristics, and structural morphology. However, low-grade graphite ores generally contain more impurities, have complex surface textures and irregular shapes, and the visual differences among ores of different grades are not always sufficiently distinct. If rapid and accurate recognition cannot be achieved at the front end of sorting, low-grade or non-conforming ores may easily enter subsequent processing stages, thereby increasing energy consumption, reagent usage, and equipment wear, while also affecting the quality of the final product. Therefore, developing an efficient graphite ore grade detection method suitable for industrial environments has important practical value for improving sorting efficiency, reducing production costs, and promoting intelligent mining.

Traditional graphite ore grade detection mainly relies on manual empirical judgment and physicochemical analytical methods. Common approaches include manual sampling, laboratory chemical analysis, X-ray diffraction (XRD), scanning electron microscopy (SEM), and carbon–sulfur analyzer testing [6,7]. These methods can provide relatively accurate results in terms of mineral composition, elemental content, and microstructure, and play an important role in ore property characterization and beneficiation process optimization. For example, Cui et al. [8] analyzed the physicochemical characteristics of flake graphite ore using XRD and electron microscopy, providing a basis for evaluating its beneficiation potential. Sader et al. [9] determined the carbon content of graphite ore using carbon–sulfur analysis, offering an effective reference for graphite ore grade evaluation. Although these methods provide high detection accuracy, their procedures are generally complex, require specialized equipment and trained personnel, and are difficult to apply in continuous, online, and real-time analysis. For the large-scale and rapid sorting requirements of mine sites, traditional methods still have evident limitations in detection efficiency, cost control, and automation level.

With the development of machine vision and machine learning technologies, intelligent recognition methods based on mineral images have gradually become an important research direction in ore sorting [10]. Compared with traditional laboratory testing methods, vision-based detection can rapidly acquire surface information from ore samples in a non-contact manner and complete category discrimination or grade recognition using algorithmic models. It therefore offers advantages such as high detection speed, a high degree of automation, and flexible deployment. Early studies typically described ore images using hand-crafted features, including color, texture, shape, edge, and spectral characteristics, and then combined these features with traditional machine learning algorithms such as support vector machines, random forests, k-nearest neighbors, or decision trees for classification. For example, Cevik et al. [11] applied machine learning methods to classify mineral resource categories, improving the consistency of mineral classification results. Pereira et al. [12] used color and texture features extracted from mineral thin-section images for mineral recognition, demonstrating the feasibility of image features in mineral classification tasks. However, such methods generally depend on manually designed features, and their performance is susceptible to illumination variation, dust contamination, background interference, and differences in ore surface characteristics. When ore targets have complex morphologies, pronounced texture variations, or only subtle differences among grades, traditional machine learning methods often struggle to achieve stable recognition performance.

In recent years, the development of deep learning has provided new solutions for mineral image analysis. Compared with traditional methods that rely on hand-crafted features, deep neural networks can directly learn multi-level and highly abstract visual features from raw images, thereby improving the representation capability of models under complex backgrounds and subtle visual differences [13]. Convolutional neural networks have been widely applied in tasks such as ore classification, mineral particle recognition [14], microscopic mineral analysis [15], and industrial defect detection. For example, Liu et al. [16] investigated ore image classification and analyzed the effects of network depth, model architecture, and data scale on recognition performance, indicating that lightweight deep models have considerable application potential in intelligent ore sorting. Relevant review studies have also shown that deep learning has gradually become an important technical foundation for mineral image recognition and segmentation, promoting the transition of mineral visual analysis from traditional feature engineering to end-to-end intelligent modeling [17,18].

In the field of object detection, deep learning models have evolved from two-stage detectors to one-stage detectors and, more recently, to end-to-end Transformer-based detectors. Faster R-CNN improves the efficiency of candidate box generation through a region proposal network and offers strong advantages in detection accuracy [19]. The YOLO series formulates object detection as a one-stage regression problem and has demonstrated outstanding performance in real-time detection tasks [20]. DETR models object detection as a set prediction problem based on the Transformer architecture, establishing an end-to-end detection framework [21]. Subsequently, Deformable DETR introduced a deformable attention mechanism to alleviate the slow convergence and insufficient small-object detection performance of the original DETR [22]. Building on this progress, RT-DETR was further optimized for real-time object detection. By incorporating an efficient hybrid encoder and a query selection mechanism, RT-DETR improves inference efficiency while maintaining detection accuracy, thereby providing a new technical pathway for industrial real-time detection tasks [23]. Recent studies have further emphasized the importance of multi-scale feature hierarchy in DETR-based object detection. Liu et al. proposed F-DETR by rethinking how multi-scale features are introduced into the DETR architecture, pointing out that directly feeding long multi-scale feature sequences into the encoder may increase computational cost and reduce detection efficiency [24]. Their work introduced a heterogeneous multi-branch structure to promote interaction between local and global features, achieving a better balance between detection accuracy and model complexity. This line of research indicates that efficient multi-scale feature organization is critical for improving DETR-based detectors, especially in tasks requiring both detailed local representation and global semantic modeling.

For graphite ore grade detection, existing studies have begun to introduce deep detection models into this field. For example, Xiang et al. [25] proposed a graphite ore grade recognition method based on Faster R-CNN, in which multi-scale feature fusion and a global attention mechanism were incorporated to enhance the model’s representation of key regions in graphite ore images. However, the two-stage detection framework still has certain limitations in terms of inference speed and edge deployment. Qiu et al. [26] proposed Stellar-YOLO, which improves the efficiency and robustness of graphite ore grade detection through a lightweight network architecture and achieves a favorable balance between accuracy and model complexity. Nevertheless, this method is still mainly based on the YOLO-series convolutional detection framework, leaving room for further improvement in modeling global dependencies and multi-scale feature interactions under complex scenarios. Sun et al. [27] applied an improved RT-DETR model to graphite ore grade recognition, demonstrating the application potential of end-to-end Transformer-based detectors in graphite ore detection. However, there remains scope for optimization in feature extraction efficiency, cross-scale information fusion, and refined bounding box regression.

In summary, although existing intelligent detection methods for graphite ore have alleviated, to some extent, the problems of low efficiency, high cost, and dependence on manual experience associated with traditional detection methods, they still face multiple challenges in real industrial environments. For example, graphite ores often exhibit complex surface textures, and the color and morphological differences among different grades may be subtle, increasing the difficulty of model discrimination. In addition, mine-site environments commonly involve uneven illumination, dust occlusion, ore overlap, background interference, and motion blur, which can easily lead to missed detections, false detections, or localization errors. Moreover, to improve detection accuracy, existing detection models often increase network depth, channel width, or attention modules, resulting in higher parameter counts and computational complexity. This increases computational cost and is unfavorable for deployment on edge devices and in real-time sorting systems. Therefore, how to reduce model complexity while maintaining high detection accuracy and further improve inference speed and localization precision remains an urgent problem in graphite ore grade detection research.

To address the above issues, and inspired by recent progress in efficient DETR architectures and multi-scale feature hierarchy optimization, this study proposes OGI-RT-DETR, a lightweight and efficient detection model for graphite ore grade recognition. The proposed model adopts RT-DETR as the baseline framework and is improved in three aspects: backbone feature extraction, neck feature fusion, and bounding box regression optimization. First, a PConv-Rep module is designed in the backbone network. By using partial convolution, this module reduces redundant computation and memory access overhead, while incorporating structural re-parameterization to improve computational efficiency during inference. This enhances the model’s ability to extract edge, texture, and irregular morphological features from graphite ore. Second, a newly designed Cross-Layer Feature Pyramid Transformer (CFPT) module is adopted to replace the original CCFM module in the neck network, thereby strengthening information interaction among features at different scales and improving the fusion of high-level semantic information with low-level details. As a result, the model can better meet the detection requirements of multi-scale ore targets under complex backgrounds. Finally, a Wise-Focaler-MPDIoU loss function is designed to further optimize the bounding box regression process by comprehensively considering sample quality, target scale, boundary shape, and internal region constraints, thereby improving localization accuracy and accelerating model convergence.

The novelty of OGI-RT-DETR does not lie in simply combining existing lightweight convolution, feature fusion, and IoU-based regression strategies. Instead, these components are integrated in a task-oriented manner according to the visual characteristics of graphite ore grade recognition. Compared with general object detection tasks, graphite ore images contain subtle inter-grade differences, irregular ore boundaries, local reflective regions, and strong background interference. Therefore, the proposed framework jointly optimizes redundant backbone computation, cross-scale semantic interaction, and boundary regression stability to address the accuracy–efficiency trade-off in real-time graphite ore sorting.

Compared with previously published graphite ore detection methods, such as Faster R-CNN-based and YOLO-based approaches, OGI-RT-DETR retains the end-to-end detection advantage of RT-DETR while further improving lightweight deployment capability. Existing studies mainly focus on enhancing detection accuracy through feature extraction or attention mechanisms, whereas the proposed model simultaneously considers fine-grained grade discrimination, inference speed, model complexity, and localization stability.

This study systematically validates the proposed model on a self-built graphite ore dataset and incorporates a simulated data augmentation strategy to improve the model’s adaptability to complex industrial environments. The experimental results show that, compared with the baseline model, OGI-RT-DETR improves mAP by 3.7%, increases FPS by 8.2%, reduces the number of parameters by 38.2%, and decreases FLOPs by 31.8%. These results indicate that the proposed method effectively reduces computational complexity while improving detection accuracy and inference speed, thereby better meeting the requirements of real-time detection and edge deployment in industrial scenarios.

The main contributions of this study are as follows:

- For complex industrial detection scenarios involving graphite ore grade recognition, a customized graphite ore image dataset is constructed and used for model training and evaluation. Meanwhile, a simulated data augmentation strategy is introduced to improve the model’s adaptability to illumination variation, background interference, and differences in ore morphology.
- A PConv-Rep backbone enhancement module is proposed, which combines the efficient computational characteristics of partial convolution with a structural re-parameterization mechanism. This module reduces redundant computation while enhancing the model’s ability to extract local texture, edge detail, and irregular morphological features from ore images.
- A CFPT feature fusion module is designed to replace the original CCFM structure, strengthening information transmission and semantic fusion among features at different scales and improving the model’s detection capability for multi-scale ore targets under complex backgrounds.
- A Wise-Focaler-MPDIoU loss function is introduced to optimize the bounding box regression process, enabling the model to focus more effectively on high-quality samples and target shape characteristics, thereby improving localization accuracy and accelerating training convergence.

## 2. Methods

### 2.1. RT-DETR-Based Detection Model

RT-DETR (Real-Time Detection Transformer) is an end-to-end detection model designed for real-time object detection tasks [28]. It improves upon the set prediction paradigm of DETR, avoiding the dependence on non-maximum suppression (NMS) post-processing commonly required by traditional detectors, while enhancing inference efficiency and detection accuracy through architectural optimization [29]. Compared with the original DETR, RT-DETR is more suitable for detection tasks in industrial scenarios that require high real-time performance.

**Figure 1:**
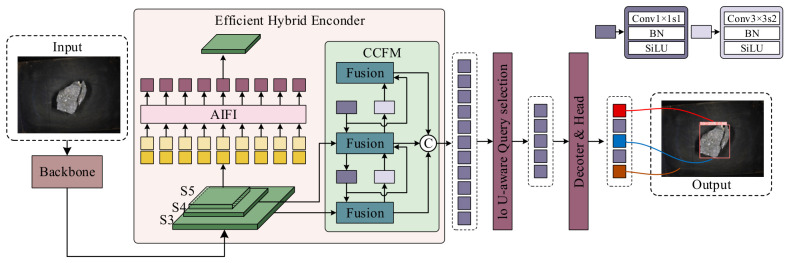
The structural framework of the RT-DETR model.

As shown in Figure 1, the overall architecture of RT-DETR mainly consists of a backbone network, an efficient hybrid encoder, an IoU-aware query selection module, and a Transformer decoder. In this study, RT-DETR-r18 is selected as the baseline model. Its backbone network adopts ResNet-18 [30] to extract multi-scale visual features and output feature maps at different levels, providing fundamental information for the subsequent encoder. In the graphite ore grade recognition task, this component is mainly responsible for extracting features such as ore color, texture, edge contours, and local reflectance characteristics.

The efficient hybrid encoder is one of the key components of RT-DETR. Its core idea is to decouple intra-scale feature interaction from cross-scale feature fusion [31]. The AIFI module is used to enhance semantic interaction within the same scale, whereas the CCFM module fuses features at different scales through convolutional operations, thereby improving feature representation quality while reducing computational overhead. Subsequently, the IoU-aware query selection mechanism selects high-quality candidate features from the encoder output as decoder queries, enabling the model to focus more effectively on regions that are likely to contain objects [32]. Finally, the Transformer decoder progressively updates the object queries layer by layer and directly outputs the category and bounding box prediction results [33].

RT-DETR-r18 achieves a favorable balance between detection accuracy and inference speed, making it suitable as the baseline model for the graphite ore grade recognition task in this study. However, graphite ore images are characterized by complex textures, irregular morphologies, subtle grade differences, and pronounced background interference. Therefore, the original RT-DETR-r18 still has room for improvement in lightweight feature extraction, fine-grained cross-scale fusion, and accurate bounding box regression. Accordingly, this study improves RT-DETR-r18 and constructs OGI-RT-DETR to further enhance detection accuracy, inference speed, and adaptability for industrial deployment.

### 2.2. Improved OGI-RT-DETR Model

**Figure 2:**
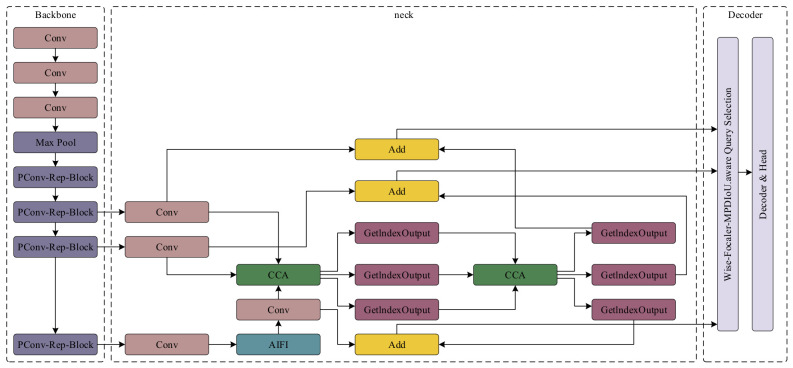
The OGI-RT-DETR model structure.

The proposed OGI-RT-DETR model adopts RT-DETR-r18 as the baseline framework and introduces improvements in three aspects: backbone feature extraction, multi-scale feature fusion in the neck, and bounding box regression optimization. The overall idea is to further enhance the model’s representation capability for fine-grained features of graphite ore while preserving the end-to-end detection advantages of RT-DETR as much as possible. At the same time, the computational complexity of the model is reduced, making it more suitable for real-time detection and edge deployment in industrial scenarios. The overall architecture of the OGI-RT-DETR graphite ore grade recognition and detection model is shown in Figure 2.

In terms of model architecture improvement, this study first uses the PConv-Rep module to replace part of the residual convolutional structures in the original backbone network of RT-DETR-r18. Partial convolution is employed to reduce redundant computation, while structural re-parameterization is incorporated to enhance feature representation during training, thereby improving the extraction of edge, texture, and irregular morphological features from graphite ore images. Second, the CFPT module is introduced into the neck network to replace the original CCFM structure. By leveraging cross-layer channel attention and cross-layer spatial attention, CFPT strengthens semantic interaction and spatial fusion among features at different scales, enabling the model to better meet the detection requirements of multi-scale ore targets under complex backgrounds. Finally, the Wise-Focaler-MPDIoU loss function is adopted to optimize the bounding box regression process, allowing the model to focus more effectively on high-quality samples, target scales, and shape characteristics, thereby improving the localization accuracy of predicted boxes and accelerating training convergence.

In summary, OGI-RT-DETR improves the efficient feature extraction capability of the backbone network through PConv-Rep, strengthens multi-scale feature fusion through CFPT, and enhances bounding box regression quality using Wise-Focaler-MPDIoU. These three improvements complement each other at the levels of feature extraction, feature fusion, and localization optimization, enabling the model to achieve a better balance among detection accuracy, inference speed, and lightweight design.

### 2.3. PConv-Rep

The original backbone network of the baseline RT-DETR-r18 model mainly adopts a ResNet structure for multi-scale feature extraction, in which the residual blocks are typically composed of consecutive 3 × 3 convolutions. This structure provides good stability and generality, but it still has certain limitations in graphite ore grade recognition. On the one hand, the effective discriminative information in graphite ore images is often concentrated in ore edges, surface textures, reflective regions, and local impurity distributions, which are characterized by strong locality and sparsity. On the other hand, industrial image acquisition environments are often affected by dark backgrounds, dust, shadows, and uneven illumination. If the backbone network performs excessive dense convolution in the shallow and intermediate stages, it may introduce redundant computation and may also weaken some fine-grained texture features [34]. Although the original backbone network has strong feature extraction capability, its consecutive dense convolutional structure involves a certain degree of computational redundancy when processing graphite ore images, and some convolutional operations contribute only limitedly to grade-discriminative features.

**Figure 3:**
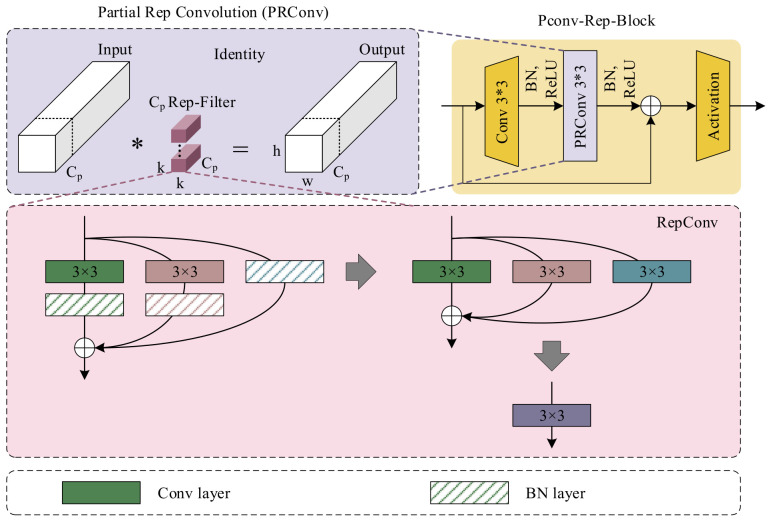
Structure of the PConv-Rep module with partial-channel convolution and bypass feature fusion.

To address this issue, the PConv-Rep module is introduced into the backbone network of RT-DETR-r18 to replace part of the convolutional structures in the original residual blocks. The structure of the PConv-Rep module is shown in Figure 3. To make the PConv mechanism clearer, the module can be understood as a partial-channel feature transformation process: the input feature channels are first divided into two groups, only one group is processed by spatial convolution, and the remaining channels are bypassed directly before being fused with the transformed channels. This module combines the low-redundancy computational advantage of partial convolution (PConv) [35] with the training enhancement and inference simplification characteristics of structural re-parameterized convolution (RepConv) [36]. While maintaining the feature map size and the number of output channels unchanged, it reduces the parameter count and computational cost of the backbone network and enhances the model’s representation capability for local textures and irregular boundaries of graphite ore.

The core idea of PConv is a channel-splitting and partial-convolution strategy: spatial convolution is performed only on a subset of the input feature channels, whereas the remaining channels preserve the original information through a direct bypass connection [37]. Let the input feature be denoted as $X\in R^{C\times H\times W}$, where C, H, and W represent the number of channels, height, and width, respectively. PConv divides the channels into two parts: one channel subset with size $c_{p}$ participates in the 3×3 convolution operation, while the other channels remain unchanged and are directly propagated to the output. If the proportion of channels involved in convolution is $r=c_{p}/C$, the main computational cost of the spatial convolution can be approximately expressed as:

(1) $${FLOPs}_{pconv}=H\times W\times K^{2}\times C_{p}^{2},$$

where K denotes the convolution kernel size, and $c_{p}$ is the number of channels involved in the convolution operation. By contrast, the computational cost of a standard 3×3 convolution is:

(2) $${FLOPs}_{conv}=H\times W\times K^{2}\times C^{2}.$$

When the partial ratio is $r=c_{p}/C=1/4$, the computational cost of the spatial convolution part in PConv is only approximately 1/16 of that of standard convolution. Although subsequent channel fusion operations are still required to ensure feature interaction, the overall computational overhead and memory access are significantly reduced. This design is well aligned with the feature distribution of the self-built graphite ore image dataset used in this study. Ore targets usually contain relatively concentrated discriminative information, whereas different channels do not contribute equally to spatial feature modeling. Therefore, it is unnecessary to repeatedly perform spatial convolution on all channels. By preserving the original responses of some channels, the model can reduce computational redundancy while mitigating information loss caused by excessive convolution, allowing ore edge contours, flaky textures, and local reflective details to maintain better stability during network propagation.

Although using PConv alone can reduce computational cost, its spatial modeling capability may be constrained by the proportion of participating channels. To further improve feature representation, this study introduces the idea of RepConv on the basis of PConv and constructs the PConv-Rep module. During training, RepConv adopts a multi-branch structure, typically including a 3×3 convolution branch, a 1×1 convolution branch, and an identity branch. The 3×3 branch captures local spatial textures, the 1×1 branch supplements inter-channel information interaction, and the identity branch preserves input features and improves gradient propagation [38,39]. This design enables the model to obtain richer feature pathways during training. During inference, the multi-branch structure can be equivalently fused into a single 3×3 convolution kernel, thereby avoiding inference latency caused by additional branches. Specifically, the equivalent convolution of RepConv can be expressed as:

(3) $$W_{Rep}=W_{3\times 3}+W_{1\times 1,pad}+W_{Identity},$$

where $W_{3\times 3}$ denotes the weight of the 3×3 convolution branch, $W_{1\times 1,pad}$ denotes the equivalent weight obtained by padding the 1×1 convolution kernel to a 3×3 size, and $W_{Identity}$ denotes the equivalent convolution weight converted from the identity branch.

After structural re-parameterization, the multi-branch structure used during training is merged into a single-path convolution during inference. Therefore, the PConv-Rep module can enhance feature learning capability during training while maintaining low computational complexity during deployment, which is particularly important for real-time ore sorting tasks on edge devices.

In the OGI-RT-DETR model, the PConv-Rep module is mainly used to replace part of the 3×3 convolutional structures in the original BasicBlock of the RT-DETR-r18 backbone network. The module first extracts basic spatial features through a standard convolution, then uses PConv-Rep to perform efficient feature transformation on part of the channels, and finally fuses the unprocessed channels with the transformed channels as the output. The residual connection is retained to ensure the stability of deep network training [40]. This design does not alter the original multi-scale output form of RT-DETR and can still provide feature maps at three scales, namely S3, S4, and S5, to the subsequent encoder. Therefore, the improved backbone network can be seamlessly integrated into the efficient hybrid encoder and decoder structure of RT-DETR.

From the perspective of task adaptation, PConv-Rep can effectively meet the dual requirements of lightweight design and strong feature representation in graphite ore grade recognition. By reducing redundant computation caused by repeated spatial convolutions in the backbone network, this module decreases the parameter count and computational complexity of the model, making it more suitable for deployment on industrial edge devices with limited computing resources. Meanwhile, the direct connection mechanism for partial channels preserves subtle information such as ore edges, particle textures, and transitions between bright and dark regions, alleviating the over-smoothing effect of consecutive convolutions on weak discriminative features. In addition, RepConv introduces a multi-branch structure during training, enhancing the model’s representation capability for complex surface morphologies. During inference, it can be equivalently transformed into a single-branch convolutional structure, thereby improving feature extraction quality while maintaining detection speed. Therefore, PConv-Rep is not merely a model compression strategy; rather, it allocates limited computational resources more effectively to the extraction and representation of key discriminative features for graphite ore grade recognition while reducing ineffective computational overhead.

In this study, the PConv-Rep module is used to replace part of the residual convolutional structures in the original RT-DETR backbone network, improving the feature extraction efficiency of the backbone without changing the overall detection framework. This design inherits the advantages of the ResNet residual structure, such as ease of training and strong stability, while combining the lightweight computation of PConv with the deployment-friendly characteristics of RepConv. It therefore provides more compact and effective multi-scale ore feature representations for the subsequent CFPT feature fusion module and detection decoder.

### 2.4. CFPT

In object detection models, the neck network is mainly responsible for fusing multi-scale features output by the backbone network, and the quality of feature fusion directly affects the classification and localization performance of the detection head. The CCFM module in the original neck network of RT-DETR mainly relies on convolutional structures for cross-scale feature fusion, offering good real-time performance and computational efficiency. However, in graphite ore grade recognition, ore targets often exhibit irregular edges, fragmented textures, obvious local reflection, and weak visual differences among different grades. If fusion relies only on conventional convolutions and scale transformations, the interaction between high-level semantic information and low-level detailed information may be insufficient, thereby affecting the model’s ability to discriminate ore contours, texture distributions, and bright–dark variations. Traditional FPN enhances multi-scale feature representation through a top-down pathway [41], while PANet further introduces a bottom-up path augmentation structure to supplement shallow localization information [42]. However, these structures usually rely on upsampling, feature concatenation, and convolutional operations, and feature blurring or semantic misalignment may occur during frequent transformations across different scales. For graphite ore grade recognition, details such as local texture, particle structure, and edge transitions are themselves important discriminative cues. Therefore, the neck network requires stronger cross-layer semantic interaction capability and fine-grained spatial modeling capability.

**Figure 4:**
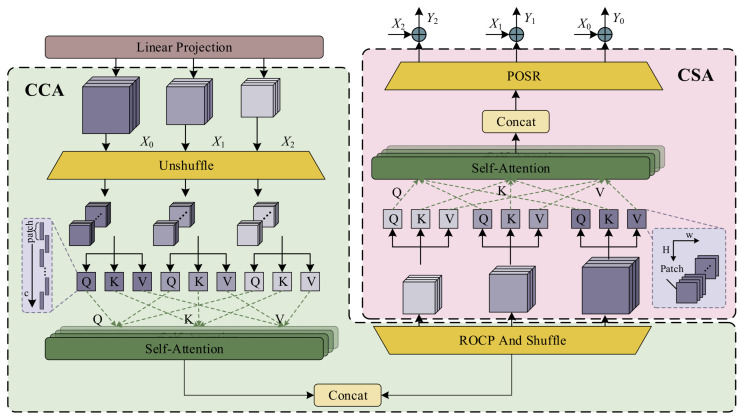
The structure of CFPT.

To address this issue, this study adopts the Cross-Layer Feature Pyramid Transformer (CFPT) [43] to replace the CCFM module in the neck network of RT-DETR. CFPT models the dependencies among features at different scales from the channel and spatial dimensions through cross-layer channel attention (CCA) and cross-layer spatial attention (CSA), respectively, thereby enhancing information interaction among multi-scale features. Compared with the original CCFM, CFPT no longer relies solely on local convolutions for scale fusion. Instead, it uses an attention mechanism to model global correlations among features at different levels, enabling the fused features to retain both high-level semantic information and low-level spatial details [44]. The structure of CFPT is shown in Figure 4.

Let the multi-scale features output by the backbone network be denoted as $\{F_{i}{\}}_{i=1}^{L}$, where L represents the number of input feature levels, and $F_{i}\in R^{C_{i}\times H_{i}\times W_{i}}$ denotes the feature map at the i-th scale. Here, $C_{i}$, $H_{i}$, and $W_{i}$ represent the number of channels, height, and width of this feature map, respectively. To facilitate cross-layer feature interaction, CFPT first aligns the channel dimensions of features at different scales through linear projection or 1×1 convolution:

(4) $$X_{i}=\phi_{i}(F_{i}),$$

where $\phi_{i}(\cdot )$ denotes the feature mapping function corresponding to the i-th scale, and $X_{i}\in R^{C_{i}\times H_{i}\times W_{i}}$ represents the feature map after channel alignment, with C being the unified channel dimension. Subsequently, CFPT sequentially establishes cross-layer channel relationships and cross-layer spatial relationships through CCA and CSA, respectively. The overall process can be expressed as:

(5) $$Y_{i}=CSA(CCA(X_{i}))+X_{i},$$

where $Y_{i}$ denotes the enhanced feature output by CFPT. The residual connection is used to preserve the original scale-specific information and improve the stability of gradient propagation during network training.

#### 2.4.1. CCA

The CCA module is mainly used to model channel dependencies among features at different levels. In graphite ore images, different channels usually respond to different types of visual information, such as ore edges, flaky textures, particle distributions, local reflections, and dark shadows. The original CCFM mainly performs channel mixing through convolution, whose modeling range is relatively local and therefore cannot fully exploit global channel correlations among features at different scales [45]. Through the cross-layer channel attention mechanism, CCA enables shallow detail features and high-level semantic features to interact sufficiently along the channel dimension, thereby enhancing the model’s representation capability for grade-discriminative features.

Specifically, CCA first performs spatial rearrangement and channel alignment on the input features $X_{i}$ at different scales, reorganizing spatial information at different resolutions into a unified channel representation. This process can be expressed as:

(6) $${\hat{X}}_{i}=R_{c}(X_{i}),$$

where $R_{c}(\cdot )$ denotes the feature rearrangement operation for channel attention calculation, and ${\hat{X}}_{i}$ represents the rearranged channel feature. Subsequently, the channel features from different scales are combined to obtain a cross-layer channel feature sequence:

(7) $$X_{c}=Concat({\hat{X}}_{1},{\hat{X}}_{2},\ldots ,{\hat{X}}_{L}).$$

On this basis, CCA generates the query matrix $Q_{c}$, key matrix $K_{c}$, and value matrix $V_{c}$, respectively:

(8) $$Q_{c}=X_{c}W_{c}^{Q},K_{c}=X_{c}W_{c}^{K},V_{c}=X_{c}W_{c}^{V},$$

where $W_{c}^{Q}$, $W_{c}^{K}$, and $W_{c}^{V}$ are learnable linear projection matrices. The cross-layer channel attention calculation can be expressed as:

(9) $$A_{c}=Softmax\left(\frac{Q_{c}K_{c}^{T}}{\sqrt{d_{c}}}\right),Z_{c}=A_{c}V_{c},$$

where $d_{c}$ denotes the channel feature dimension, $A_{c}$ represents the cross-layer channel attention weights, and $Z_{c}$ denotes the feature enhanced by channel relationships. Finally, the enhanced channel features are restored to the original pyramid structure through an inverse rearrangement operation:

(10) $${\tilde{X}}_{i}=R_{c}^{-1}(Z_{c}),$$

where $R_{c}^{-1}(\cdot )$ denotes the inverse operation of channel rearrangement, and ${\tilde{X}}_{i}$ represents the i-th level feature enhanced by CCA. Through the above process, CCA can establish more sufficient channel dependencies among features at different scales. For graphite ore grade recognition, this module enables the model to integrate high-level semantic information while focusing on local textures, avoiding the problem that shallow features only retain edges and textures but lack category-discriminative semantics. CCA not only enhances the model’s ability to extract surface texture features from ore images, but also further improves its capability to model the associations between texture features and grade categories.

#### 2.4.2. CSA

The CSA module is mainly used to model spatial correlations among features at different levels [46]. Graphite ore targets in images usually exhibit irregular block-like structures, and their boundaries may contain protrusions, depressions, and occlusions. In addition, ore surfaces are easily affected by dust, shadows, and illumination reflection. If features at different scales are insufficiently fused in the spatial dimension, the model may suffer from boundary localization deviation, insufficient response in local regions, or enhanced interference from background noise. Therefore, after channel relationship modeling, it is necessary to further introduce CSA to model cross-layer spatial information.

CSA first divides the features ${\tilde{X}}_{i}$ output by CCA into spatial patch sequences to establish spatial positional relationships among different levels. This process can be expressed as:

(11) $$P_{i}=R_{s}({\tilde{X}}_{i}),$$

where $R_{s}(\cdot )$ denotes the spatial patch partition operation, and $P_{i}$ represents the spatial patch feature corresponding to the i-th scale. Subsequently, spatial patches from different scales are combined across layers:

(12) $$X_{s}=Concat(P_{1},P_{2},\ldots ,P_{L}).$$

Similar to CCA, CSA generates the query, key, and value in the spatial dimension through linear mappings:

(13) $$Q_{s}=X_{s}W_{s}^{Q},K_{s}=X_{s}W_{s}^{K},V_{s}=X_{s}W_{s}^{V},$$

where $W_{s}^{Q}$, $W_{s}^{K}$, and $W_{s}^{V}$ are learnable parameters in CSA. The cross-layer spatial attention calculation is given by:

(14) $$A_{s}=Softmax\left(\frac{Q_{s}K_{s}^{T}}{\sqrt{d_{s}}}\right),\;Z_{s}=A_{s}V_{s},$$

where $d_{s}$ denotes the spatial feature dimension, $A_{s}$ represents the cross-layer spatial attention weights, and $Z_{s}$ denotes the feature enhanced by spatial relationships. Finally, the original feature map structure is restored through an inverse spatial patch reorganization operation:

(15) $$Y_{i}=R_{s}^{-1}(Z_{s}),$$

where $R_{s}^{-1}(\cdot )$ denotes the inverse process of spatial patch reorganization, and $Y_{i}$ is the final enhanced feature at the i-th level output by CFPT.

The role of CSA is to enable features at different scales to interact more sufficiently in terms of spatial position. For graphite ore images, shallow features usually contain rich edge and texture information, whereas high-level features have stronger category semantics and global region perception. CSA associates this type of information in the spatial dimension, enabling the model to more accurately localize ore contour regions and reduce interference from dark backgrounds, dust particles, and local reflections. Compared with simple upsampling and concatenation, CSA places greater emphasis on adaptive selection of effective spatial regions, thereby improving the robustness of the model for ore targets in complex scenarios.

In summary, CFPT performs cross-layer channel modeling and cross-layer spatial modeling through CCA and CSA, respectively, enabling the neck network to more fully fuse the multi-scale features output by the backbone network. CCA strengthens channel semantic interaction among different scales, allowing the model to better capture discriminative information such as ore texture, reflection, and particle structure. CSA enhances the expression of cross-layer spatial relationships and improves the model’s perception of ore boundaries, morphology, and local regions. By replacing the original CCFM module with CFPT, the proposed method effectively alleviates semantic misalignment and detail loss in conventional scale fusion, providing more complete, stable, and discriminative multi-scale feature representations for the subsequent decoder. This further improves the detection accuracy and robustness of OGI-RT-DETR in graphite ore grade recognition.

### 2.5. Wise-Focaler-MPDIoU

In object detection tasks, the bounding box regression loss directly affects the fitting quality between predicted boxes and ground-truth boxes and is one of the key factors determining localization accuracy. The original RT-DETR model generally uses IoU-related losses to constrain bounding box regression. Such methods can evaluate the quality of predicted boxes from the perspective of regional overlap, but they still have certain limitations in complex scenarios. For graphite ore grade recognition, ore targets often exhibit irregular shapes, rough boundaries, uneven surface reflections, and low contrast between the background and local ore regions. When edge offsets or corner misalignment occur between predicted boxes and ground-truth boxes, optimization based only on overlap area may not fully reflect subtle geometric errors in the predicted box. Especially under blurred ore boundaries or partial occlusion, the model is prone to producing localization boxes that are too large, too small, or insufficiently fitted to the target.

To further improve bounding box regression accuracy, this study introduces the Wise-Focaler-MPDIoU loss function to optimize the original bounding box regression loss. This loss integrates the advantages of MPDIoU [47], Focaler-IoU [48], and Wise-IoU [49]. MPDIoU enhances the geometric alignment capability of bounding boxes by minimizing the distances between the corresponding corner points of the predicted and ground-truth boxes. Focaler-IoU adjusts the optimization weights of samples with different qualities through an interval mapping mechanism, enabling the model to focus more on effective regression samples. Wise-IoU introduces a dynamic focusing mechanism that adaptively adjusts gradient contributions according to sample regression quality, thereby reducing the interference of abnormal samples and low-quality annotations during training. By combining these three components, the model can not only improve the overlap between predicted boxes and ground-truth boxes, but also further optimize the fitting of ore boundary corners and contour regions.

Let the predicted box be $B=(x_{1},y_{1},x_{2},y_{2})$, and the ground-truth box be $B^{gt}=(x_{1}^{gt},y_{1}^{gt},x_{2}^{gt},y_{2}^{gt})$. The intersection over union (IoU) between them is defined as:

(16) $$IoU=\frac{\left|B\cap B^{gt}\right|}{\left|B\cup B^{gt}\right|},$$

where $\left|B\cap B^{gt}\right|$ denotes the intersection area between the predicted box and the ground-truth box, and $\left|B\cup B^{gt}\right|$ denotes their union area. The conventional IoU loss can be expressed as:

(17) $$L_{IoU}=1-IoU,$$

This loss reflects the overlap relationship between the predicted and ground-truth boxes. However, when two bounding boxes have similar overlap areas but different corner positions, or when there is a significant shape deviation between the predicted and ground-truth boxes, IoU cannot sufficiently characterize localization errors. Therefore, this study further introduces MPDIoU as a geometric constraint term.

MPDIoU starts from the geometric representation of horizontal bounding boxes and models a rectangular box as a spatial region jointly constrained by the upper-left and lower-right corner points. Therefore, compared with considering only the center-point distance or the width–height ratio, directly constraining the distances between two corresponding corner points can more comprehensively describe the geometric deviation between the predicted and ground-truth boxes. Let the squared distance between the upper-left corner points of the predicted and ground-truth boxes be $d_{1}^{2}$, and the squared distance between the lower-right corner points be $d_{2}^{2}$. Then:

(18) $$d_{1}^{2}=(x_{1}-x_{1}^{gt}{)}^{2}+(y_{1}-y_{1}^{gt}{)}^{2},$$

(19) $$d_{2}^{2}=(x_{2}-x_{2}^{gt}{)}^{2}+(y_{2}-y_{2}^{gt}{)}^{2},$$

MPDIoU can be defined as:

(20) $$MPDIoU=IoU-\frac{d_{1}^{2}}{w^{2}+h^{2}}-\frac{d_{2}^{2}}{w^{2}+h^{2}},$$

where w and h denote the width and height of the boundary region used for normalization, respectively. The corresponding MPDIoU loss is:

(21) $$L_{MPDIoU}=1-MPDIoU.$$

From the above definition, MPDIoU considers both the overlap area and corner-point distances. When the predicted box has a high overlap with the ground-truth box but its corner points are still misaligned, MPDIoU imposes an additional penalty, thereby encouraging the predicted box to further approach the true ore boundary. For graphite ore targets with irregular edges and large morphological variations, this constraint can improve the fitting degree between the detection box and the ore contour.

Although MPDIoU can enhance geometric localization capability, the regression difficulty varies among samples during training. Some samples have clear boundaries and complete targets, making them relatively easy for the model to learn. In contrast, other samples are affected by shadows, dust, reflections, or background interference, making bounding box regression more difficult. If all samples participate in optimization in the same manner, the model may consume excessive gradient resources on a large number of easy samples while paying insufficient attention to difficult samples that truly affect detection performance. Therefore, this study introduces the idea of Focaler-IoU to perform interval remapping for regression samples. Let s denote the IoU or MPDIoU metric value. The Focaler mapping function can be expressed as:

(22) $$Focaler(s)=\left\{\begin{array}{ll} 0, & s<d\\ \frac{s-d}{u-d}, & d\leq s\leq u\\ 1, & s>u \end{array}\right.,$$

where d and u denote the lower and upper bounds of interval mapping, respectively, satisfying 0≤d<u≤1. This mapping adjusts the regression attention range according to sample quality, enabling the model to focus more on optimizing samples within the effective interval. By combining the Focaler mechanism with MPDIoU, the Focaler-MPDIoU loss can be obtained as:

(23) $$L_{Focaler-MPDIoU}=1-Focaler(MPDIoU),$$

This loss further enhances the learning capability for effective difficult samples while maintaining the corner-distance constraint. For graphite ore grade recognition, this design helps the model pay more attention to ore samples with blurred boundaries, local reflections, or strong texture interference, rather than repeatedly optimizing only easily localized samples.

On this basis, this study further introduces the dynamic focusing mechanism of Wise-IoU. The core idea of Wise-IoU is to dynamically allocate gradient gains according to sample regression quality, preventing low-quality abnormal samples from producing excessive negative gradients while also reducing the occupation of training resources by well-regressed samples. Let the regression loss of the current sample be $L_{MPDIoU}$, and its moving average be ${\bar{L}}_{MPDIoU}$. The outlier degree of the sample can be expressed as:

(24) $$\beta =\frac{L_{MPDIoU}}{{\bar{L}}_{MPDIoU}},$$

where β is used to measure the regression quality of the current sample relative to the overall training state. The Wise dynamic focusing coefficient is further defined as:

(25) $$\omega (\beta )=\frac{\beta }{\delta \alpha^{\beta -\delta }},$$

where α and δ are hyperparameters controlling the intensity of dynamic focusing. This weight performs non-monotonic adjustment for samples of different qualities, enabling the model to focus more on samples with effective learning value instead of blindly amplifying the gradient influence of extremely difficult samples or abnormal annotations.

Finally, the Wise-Focaler-MPDIoU loss function adopted in this study is defined as:

(26) $$L_{Wise-Focaler-MPDIoU}=\omega (\beta )\cdot L_{Focaler-MPDIoU}.$$

In terms of the optimization mechanism, Wise-Focaler-MPDIoU simultaneously considers three factors. MPDIoU enhances the geometric alignment between predicted and ground-truth boxes, enabling the model to fit ore boundaries more accurately. The Focaler mechanism adjusts the sample attention range through interval mapping, encouraging the model to focus more on regression samples that make practical contributions to detection performance. The Wise dynamic focusing mechanism adaptively assigns gradient weights according to sample quality, reducing the influence of abnormal and low-quality samples on training stability. Together, these components allow the bounding box regression process to optimize not only the overlap area, but also the overlap relationship, corner positions, and sample learning weights.

For the graphite ore grade recognition task in this study, Wise-Focaler-MPDIoU is highly applicable. On the one hand, graphite ore usually exhibits irregular block-like structures with non-smooth boundaries, and the corner-distance constraint can improve the fitting degree between predicted boxes and ore contours. On the other hand, the differences in color, texture, and reflection among ores of different grades are subtle, and localization errors may further affect subsequent category discrimination. By introducing dynamic focusing and difficult-sample reweighting mechanisms, the model can learn the boundary features of complex samples more stably during training, thereby reducing missed detections, false detections, and localization deviations. Therefore, replacing the original bounding box regression loss with Wise-Focaler-MPDIoU helps improve the localization accuracy and detection robustness of OGI-RT-DETR in complex industrial scenarios.

## 3. Data and Experimental Preparation

### 3.1. Data Collection

The graphite ore image dataset used in this study was obtained from real mine acquisition scenarios. The samples were collected from mining areas related to China Minmetals Group (Heilongjiang) Graphite Industry Co., Ltd., located in Hegang City, Heilongjiang Province, China. To ensure good data representativeness, various graphite ore samples were selected from different sampling areas under the guidance of on-site professionals. The sample types included both primary graphite ore and oxidized graphite ore. This was intended to cover, as comprehensively as possible, the common appearance variations in ores in actual production environments, thereby providing a more reliable data basis for subsequent model training.

Image acquisition was conducted under visible-light conditions using a Hikrobot MV-CA050-12GC industrial camera (Hangzhou Hikrobot Co., Ltd., Hangzhou, Zhejiang, China). The camera was fixed at a relatively stable shooting position, and ore samples were photographed under the actual illumination conditions of the beneficiation site. To reduce information bias caused by a single viewing angle, each ore sample was imaged from different angles and different surface regions, allowing the dataset to more comprehensively reflect the morphology, texture, color, and reflection characteristics of the ore. All original images were saved in JPG format, with the image resolution uniformly set to 640×640 pixels for subsequent annotation, augmentation, and model training. Finally, a graphite ore grade recognition dataset containing 1259 high-quality original images was constructed. It should be noted that the image acquisition in this study was conducted in a real beneficiation-site environment but under a relatively controlled static shooting condition. The dataset therefore reflects real ore appearances, illumination variations, surface oxidation, texture differences, and background interference to a certain extent. However, it does not fully represent continuous conveyor-belt sorting conditions, where ore overlap, motion blur, dense target distribution, and rapidly changing illumination may occur. Therefore, the experiments in this study should be regarded as offline validation based on real-mine static images, rather than full online verification in a continuous industrial sorting system.

The grade categories of graphite ore were mainly determined based on the measured carbon content of the samples and were further verified using the experience of on-site experts. According to differences in fixed carbon content, the samples were divided into three categories: low-grade, medium-grade, and high-grade graphite ore. Specifically, low-grade graphite ore had a carbon content of 0–10%, medium-grade graphite ore had a carbon content of 10–20%, and high-grade graphite ore had a carbon content higher than 20%. The carbon-content thresholds used in this study were determined according to the grade classification practice of the sampled mining area and the requirements of subsequent graphite ore sorting experiments. In practical production, ores with a fixed carbon content below 10% are usually regarded as low-grade materials with limited direct economic value, ores with a carbon content of 10–20% are considered medium-grade materials requiring further beneficiation, and ores with a carbon content above 20% are treated as relatively high-grade materials with higher sorting priority. This threshold setting was also checked and confirmed by on-site professionals based on measured carbon content and production experience. Therefore, the three-grade classification scheme used in this study reflects both the measured carbon-content differences and the practical needs of graphite ore sorting.

From the perspective of visual characteristics, graphite ores of different grades exhibit certain differences. In general, high-grade ore has a higher graphite content and therefore appears darker overall, with relatively concentrated surface gloss and texture distributions. Low-grade ore contains more light-colored minerals such as quartz and silicates, usually showing a brighter appearance and more pronounced mottled surface patterns. Medium-grade ore lies between the two, with transitional characteristics in color and texture. In addition, oxidized graphite ore may be affected by associated minerals such as hematite and pyrite, causing some local regions to exhibit reddish-brown or light-yellow features. These complex appearances indicate that graphite ore grade recognition is not a simple color classification problem. Instead, the model must simultaneously consider multiple types of visual information, including ore edge morphology, surface texture, local reflection, and color distribution.

**Figure 5:**
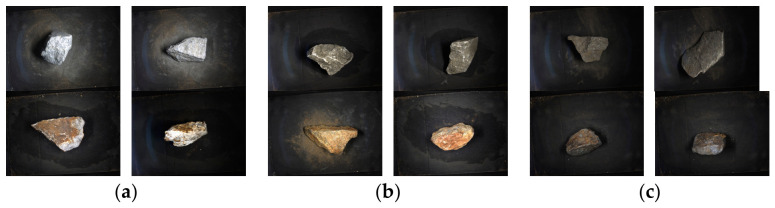
Representative images from the graphite ore dataset. The upper row presents primary graphite ore samples, whereas the lower row presents oxidized graphite ore samples: (**a**) Low-grade graphite ore (0–10%); (**b**) Medium-grade graphite ore (10–20%); (**c**) High-grade graphite ore (above 20%).

Examples of images from the graphite ore dataset constructed in this study are shown in Figure 5. The dataset was acquired from real mining environments and contains graphite ore samples of different types, grades, and appearance states. These images provide a useful basis for evaluating the model’s ability to recognize grade-related visual features, such as ore morphology, surface texture, local reflection, and subtle inter-grade differences. However, because the samples are mainly static and relatively isolated, they cannot fully reproduce the dynamic conditions of conveyor-belt sorting. Training and testing the model on this dataset can therefore provide preliminary validation of OGI-RT-DETR for graphite ore grade recognition, while further online experiments in continuous industrial sorting environments are still required.

### 3.2. Data Processing

To ensure that the dataset met the training requirements of the object detection model, the graphite ore images were annotated, augmented, and divided into subsets after the original image acquisition. During practical image acquisition, graphite ore images are easily affected by illumination variation, background interference, differences in ore posture, and surface reflection. If only the original images are used for training, the model may become highly dependent on specific acquisition conditions and exhibit insufficient generalization capability. Therefore, while preserving the original ore texture and grade-related characteristics, this study introduced several data augmentation methods to expand sample diversity and simulate imaging variations under complex industrial environments. It should be noted that although the ore samples were collected from real mining scenarios, the images used in this study mainly present relatively isolated and static ore targets rather than continuous conveyor-belt scenes. This acquisition setting helps reduce uncontrolled interference and allows the model to focus on grade-related visual features, such as color, texture, edge contours, and surface morphology. However, it cannot fully represent practical conveyor-belt sorting conditions, where ore overlap, partial occlusion, motion blur, continuous background changes, and dynamic illumination may occur. Therefore, the dataset in this study should be regarded as a controlled real-mine image dataset for validating the feasibility of graphite ore grade recognition, rather than a complete simulation of all online sorting conditions.

In this study, the LabelImg [50] image annotation tool was used to manually annotate the graphite ore images. During annotation, the visible boundary of each ore target was used as the range of the bounding rectangle, and the label was assigned according to the grade category corresponding to the measured carbon content of the sample. The dataset included three categories: low-grade (0–10%), medium-grade (10–20%), and high-grade (20%+). The annotation files were saved as TXT files in YOLO format. This format records the target category index, bounding box center coordinates, width, and height, allowing the data to be directly used for subsequent detection model training.

**Figure 6:**
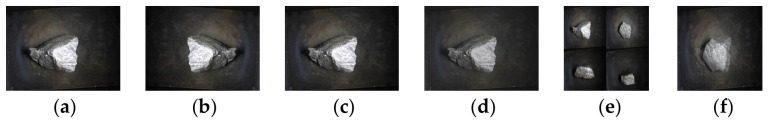
Visual comparison of data augmentation results for graphite ore images: (**a**) Original Image; (**b**) Random horizontal flip; (**c**) Median filtering for denoising; (**d**) Contrast adjustment; (**e**) Mosaic data augmentation; (**f**) MixUp data augmentation.

To improve the adaptability of the model to complex scenarios, the following five data augmentation strategies were adopted in this study. (i) Random horizontal flipping: images were flipped left to right to simulate the visual states of ores placed in different orientations. Since graphite ore has no fixed orientation, this operation does not change the category semantics and can enhance the model’s adaptability to target orientation variations. (ii) Median filtering for denoising: median filtering was used to suppress random noise and local interference in the images while preserving ore edges and texture information as much as possible, thereby improving the robustness of the model under complex acquisition environments. (iii) Contrast adjustment: image contrast was randomly adjusted within a certain range to simulate variations in illumination intensity, shadows, and reflections in the field, enabling the model to adapt to ore images under different brightness conditions. (iv) Mosaic data augmentation: four images were randomly selected and stitched together to generate a new composite sample, thereby increasing the diversity of target scales, spatial positions, and background combinations and improving the model’s detection capability for multi-scale ore targets. (v) MixUp data augmentation: two images and their annotation information were linearly blended to generate mixed samples, which helps alleviate model overfitting and enhances generalization to samples with blurred boundaries, similar textures, and local interference. The comparative effects of graphite ore data augmentation are shown in Figure 6. The adopted augmentation strategies mainly aim to simulate variations in illumination, noise interference, ore posture, background composition, target scale, and boundary ambiguity. Among them, Mosaic and MixUp can partially increase the diversity of spatial combinations and sample mixing, which provides limited simulation of complex target distributions. Nevertheless, these augmentation methods cannot fully reproduce the dynamic characteristics of conveyor-belt sorting, such as motion blur caused by belt movement, dense ore accumulation, object-to-object occlusion, and continuous frame-to-frame background variation. This limitation was considered when interpreting the experimental results, and further validation using conveyor-belt images or video data will be necessary before large-scale industrial deployment.

To avoid data leakage, the original images were first divided into training, validation, and test sets at a ratio of 7:2:1 before data augmentation. Data augmentation was then applied only to the training set, while the validation and test sets were kept independent and were not augmented for model evaluation. This strategy ensured that augmented versions of the same original image did not appear across different subsets. The training set was used for model parameter learning, the validation set was used for performance monitoring and parameter tuning during training, and the test set was used for the final evaluation of detection performance. After data augmentation and annotation organization, a dataset containing 4216 graphite ore instances was finally obtained, including 1704 low-grade, 1669 medium-grade, and 843 high-grade samples. This distribution effectively reflects the imbalance in the number of samples among different grades during practical graphite ore sorting and makes model training more consistent with real industrial application scenarios. Details of the dataset partition are shown in Table 1.

Through standardized annotation, multi-strategy data augmentation, and reasonable dataset partitioning, this study established an experimental data foundation suitable for graphite ore grade detection. This processing workflow not only increased the number of samples and the diversity of scenarios, but also improved the model’s adaptability to illumination variation, background interference, ore posture changes, and subtle grade differences. It therefore provided reliable support for the subsequent training and performance validation of the OGI-RT-DETR model under the current controlled real-mine image setting.

### 3.3. Experimental Environment

To ensure the validity and reproducibility of the experimental results, the proposed OGI-RT-DETR model for graphite ore grade recognition and detection was trained using a unified set of parameters. The input image size was set to 640×640, the initial learning rate was set to 0.001, the batch size was set to 4, and the number of data loading workers was set to 4. The entire training process was conducted for 200 epochs. Network parameters were optimized using the stochastic gradient descent (SGD) optimizer, with the momentum parameter set to 0.09 to improve training stability and convergence efficiency. All experiments were conducted on a workstation equipped with a 12th Gen Intel Core i5-12490F CPU (3.00 GHz, Intel Corporation, Ho Chi Minh City, Vietnam) and an NVIDIA GeForce RTX 3080 Ti GPU (NVIDIA Corporation, Santa Clara, CA, USA). The detailed experimental environment configuration is shown in Table 2.

### 3.4. Evaluation Metrics

To comprehensively evaluate the detection performance of OGI-RT-DETR in graphite ore grade recognition, this study assessed the model from three aspects: detection accuracy, inference speed, and lightweight performance. Detection accuracy was mainly evaluated using Precision, Recall, and mAP50; inference speed was measured by FPS; and model complexity was analyzed using FLOPs and Params. These metrics provide an intuitive reflection of the model’s overall performance in terms of detection accuracy, real-time capability, and deployment feasibility.

Precision (P) represents the proportion of correctly predicted positive samples among all targets predicted as positive by the model, and is mainly used to evaluate false detections. A higher Precision indicates that the model is less likely to incorrectly identify the background or ores of other grades as the target category. It is calculated as follows:

(27) $$Precision=\frac{TP}{TP+FP}\times 100\%.$$

Recall (R) represents the proportion of correctly detected positive samples among all actual positive samples, and mainly reflects missed detections. A higher Recall indicates that the model can detect more truly existing graphite ore targets. It is calculated as follows:

(28) $$Recall=\frac{TP}{TP+FN}\times 100\%,$$

where TP denotes the number of correctly detected positive samples, FP denotes the number of samples incorrectly predicted as positive, and FN denotes the number of actual positive samples that are not detected by the model. For graphite ore grade recognition, Precision and Recall correspond to the model’s false-detection control capability and target detection capability, respectively, and both should be considered comprehensively.

Mean average precision (mAP) is a commonly used comprehensive evaluation metric in object detection tasks. It measures the overall performance of a model in multi-class detection by calculating the mean of the AP values across all categories. The dataset used in this study contains three categories of graphite ore, namely low-grade, medium-grade, and high-grade ore; therefore, the number of classes is N = 3. When the intersection over union (IoU) threshold between the predicted box and the ground-truth box is set to 0.5, mAP is denoted as mAP50, and its calculation is given by:

(29) $${mAP}_{50}=\frac{1}{N}{\sum_{c=1}^{N}AP}_{c}^{0.5},$$

where ${AP}_{c}^{0.5}$ denotes the average precision of the c-th category at an IoU threshold of 0.5, and N denotes the total number of categories. mAP50 comprehensively reflects the detection performance of the model in terms of category recognition and object localization and is the core metric used in this study to evaluate detection accuracy.

FPS is used to measure the number of image frames that the model can process per second, and is an important metric for evaluating inference speed and real-time detection capability [51]. A higher FPS indicates faster detection speed under the same hardware conditions, making the model more suitable for real-time scenarios such as ore conveyor-belt sorting and online detection. Since industrial environments are usually sensitive to detection latency, FPS is an important reference for determining whether a model can be practically deployed.

FLOPs are used to measure the number of floating-point operations required by the model during a single forward inference process, reflecting the computational complexity of the model. Taking convolution as an example, the computational cost can be expressed as:

(30) GFLOPS=W×H×K×K×Cin×Cout,

where W and H denote the width and height of the input feature map, respectively; K denotes the convolution kernel size; Cin denotes the number of input channels; and Cout denotes the number of output channels. Lower FLOPs indicate lower computational overhead, which is more advantageous for operation on edge devices or platforms with limited computing resources.

Params denotes the total number of trainable parameters in the model and directly affects storage occupation and deployment cost [52]. In general, fewer parameters indicate lower requirements for GPU memory and storage resources, facilitating deployment on industrial field devices. Therefore, when evaluating OGI-RT-DETR, this study considers mAP50, FPS, FLOPs, and Params simultaneously to comprehensively determine whether the model achieves a favorable balance among detection accuracy, inference speed, and lightweight performance.

## 4. Results and Discussion

### 4.1. Model Training and Confusion Matrix Visualization

In this study, comparative experiments with the baseline RT-DETR-r18 model were conducted to verify the detection performance and improvement effects of OGI-RT-DETR. The confusion matrix provides an intuitive representation of the correspondence between predicted categories and ground-truth categories. It not only reflects the correct recognition proportion of each category, but also reveals misclassification among ores of different grades, thereby helping analyze the strengths and limitations of the model in category discrimination.

**Figure 7:**
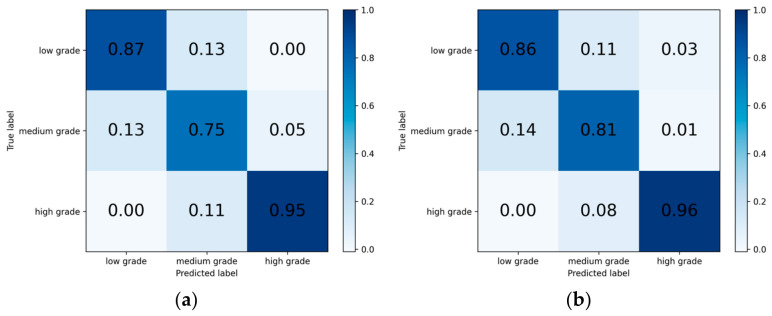
Confusion matrix visualization of different models: (**a**) Confusion matrix obtained using the baseline RT-DETR model; (**b**) Confusion matrix obtained using the proposed OGI-RT-DETR model.

To further analyze the recognition performance for different grade categories, the confusion matrices of the two models were plotted, as shown in Figure 7. The confusion matrix intuitively reflects the correspondence between model predictions and ground-truth labels [53]. Higher values on the diagonal indicate more accurate recognition of the corresponding category, whereas off-diagonal values indicate misclassification among categories.

As shown in Figure 7a, the original RT-DETR-r18 achieves correct recognition proportions of 0.87, 0.75, and 0.95 for low-grade, medium-grade, and high-grade ores, respectively. Among them, the recognition performance for medium-grade ore is relatively weaker, mainly because its visual characteristics lie between those of low-grade and high-grade ores and are easily affected by similar colors, indistinct texture differences, and local reflections. As shown in Figure 7b, OGI-RT-DETR improves the correct recognition proportion of medium-grade ore from 0.75 to 0.81 and that of high-grade ore from 0.95 to 0.96, indicating that the improved model enhances discrimination for both easily confused categories and high-grade categories. Although the correct recognition proportion of low-grade ore slightly decreases from 0.87 to 0.86, the proportion of low-grade ore misclassified as medium-grade ore decreases from 0.13 to 0.11, suggesting that the confusion between low-grade and medium-grade ores is alleviated to some extent.

Overall, OGI-RT-DETR improves the recognition of fine-grained features in graphite ore by enhancing feature extraction, multi-scale fusion, and bounding box regression capabilities. The confusion matrix results further demonstrate that the improved model outperforms the original RT-DETR-r18 in terms of overall detection accuracy and category discrimination capability.

**Figure 8:**
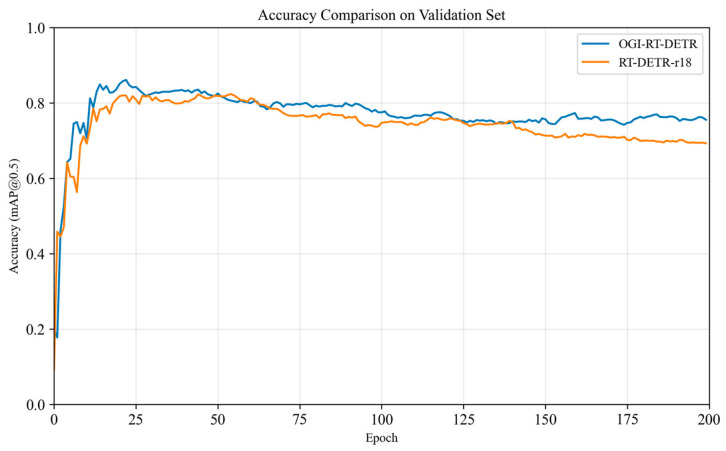
Validation accuracy curves of the proposed OGI-RT-DETR model and the baseline RT-DETR model.

As shown in Figure 8, the improved OGI-RT-DETR model and the baseline RT-DETR-r18 model were trained on the augmented graphite ore dataset using the same predefined parameters. In the early training stage, the mAP50 values of both models increased rapidly, indicating that both models could quickly learn basic visual features from graphite ore images. Among them, OGI-RT-DETR showed a more pronounced improvement and entered a higher-accuracy range earlier, suggesting that the improved architecture helps enhance the model’s early-stage feature learning capability. In the middle stage of training, the two curves generally became stable; however, OGI-RT-DETR maintained higher accuracy than RT-DETR-r18 in most epochs, indicating its advantages in multi-scale feature representation and category discrimination. In the later training stage, the curve of RT-DETR-r18 showed a certain decline, whereas OGI-RT-DETR exhibited smaller fluctuations and more stable overall convergence. Finally, RT-DETR-r18 achieved an mAP50 of 82.4%, while OGI-RT-DETR reached an mAP50 of 86.1%, representing an improvement of 3.7 percentage points. This indicates that the improved model can more effectively learn fine-grained features of graphite ore and enhance the accuracy and stability of grade detection.

### 4.2. Ablation Experiments

To verify the actual contribution of each proposed improvement module to the performance enhancement of OGI-RT-DETR, ablation experiments were conducted using RT-DETR-r18 as the baseline model under the same dataset, training parameters, and experimental environment. The experiments were performed in a stepwise accumulation manner, in which different improvement strategies were introduced into the model separately to analyze the independent effects and combined performance of each module. The comparative results of the ablation experiments are shown in Table 3. The improvement strategies in this study mainly include the following three components:

- A: The PConv-Rep module is used to replace the original backbone network, reducing redundant computation while enhancing the model’s ability to extract local textures, edge details, and irregular morphological features of graphite ore.
- B: The CFPT feature fusion module is adopted to replace the original CCFM structure, strengthening information transmission and semantic fusion among features at different scales and improving the model’s detection capability for ore targets under complex backgrounds and at multiple scales.
- C: The Wise-Focaler-MPDIoU loss function is introduced to optimize the bounding box regression process, enabling the model to focus more effectively on high-quality samples and target shape characteristics, thereby improving localization accuracy and accelerating training convergence.

The baseline RT-DETR-r18 achieves P, R, mAP50, and FPS values of 83.2%, 85.7%, 82.4%, and 78.1, respectively. When only strategy A is introduced, the mAP50 of the model increases to 83.7%, and FPS increases to 82.6. Meanwhile, FLOPs decrease from 58.2 G to 44.1 G, and Params decrease from 20.05 M to 14.25 M. This indicates that PConv-Rep can effectively reduce model complexity while maintaining and improving the backbone network’s ability to extract key features from graphite ore. When strategy B is introduced alone, mAP50 reaches 84.2%, indicating that CFPT has a significant positive effect on cross-scale feature fusion and can alleviate recognition difficulties caused by feature similarity among ores of different grades. When only strategy C is adopted, mAP50 increases to 83.4%, suggesting that the improved loss function can optimize the bounding box regression process and improve model localization accuracy to some extent.

Further observation of the combined strategies shows that the modules have good synergistic effects. When A and B are introduced simultaneously, the model’s mAP50 increases to 85.3%, FPS reaches 84.7, and FLOPs and Params decrease to 39.8 G and 12.42 M, respectively. This indicates that combining the lightweight backbone network with the cross-layer feature fusion module can improve detection accuracy while reducing computational overhead. The A+C and B+C combinations also achieve mAP50 values of 84.6% and 85.0%, respectively, further demonstrating that loss function optimization can complement structural improvements.

Finally, after introducing A+B+C, the complete OGI-RT-DETR model achieves P, R, mAP50, and FPS values of 85.6%, 87.6%, 86.1%, and 84.5, respectively. Compared with the baseline model, mAP50 increases by 3.7 percentage points, FPS improves by approximately 8.2%, and FLOPs and Params decrease by approximately 31.8% and 38.2%, respectively. These results indicate that the three proposed improvement modules are not simply stacked independently, but effectively complement each other at the levels of feature extraction, feature fusion, and bounding box regression. As a result, the model achieves a better balance among detection accuracy, inference speed, and lightweight performance.

**Table 3:** Comparison of indicators of ablation experiments.

Methods	P (%)	R (%)	mAP50 (%)	FPS	FLOPS (G)	Params (M)
RT-DETR-r18	83.2	85.7	82.4	78.1	58.2	20.05
A	84.1	86.2	83.7	82.6	44.1	14.25
B	83.7	86.5	84.2	80.8	53.6	18.24
C	83.9	85.9	83.4	78.3	58.3	20.08
A+B	86.2	87.3	85.3	84.7	39.8	12.42
A+C	84.7	86.8	84.6	82.4	44.3	14.26
B+C	85.1	87.0	85.0	80.6	53.7	18.22
OGI-RT-DETR (A+B+C)	85.6	87.6	86.1	84.5	39.7	12.39

To further analyze the practical influence of each improved module on graphite ore grade detection performance, this study used RT-DETR-r18 as the baseline framework. Under identical training conditions, the backbone network, neck network, and bounding box regression loss function were replaced and combined for verification. The ablation results of each module are shown in Table 4.

In the comparison of bounding box regression losses, Wise-IoU, Focaler-IoU, and MPDIoU all improve model detection performance to some extent, achieving mAP50 values of 82.8%, 83.0%, and 83.1%, respectively. This indicates that dynamic sample weighting, hard-sample focusing, and geometric distance constraints all have positive effects on graphite ore localization. In comparison, Wise-Focaler-MPDIoU further increases mAP50 to 83.4%, with P and R reaching 83.9% and 85.9%, respectively, while FPS remains at 78.3. These results show that the proposed loss function can enhance bounding box regression quality without substantially increasing computational burden. For graphite ore targets with irregular edges, obvious reflective regions, and subtle texture differences, this loss function can better constrain the geometric relationship between predicted boxes and ground-truth boxes, thereby improving localization stability.

In the comparison of backbone modules, Faster-Block, DualConv, and PConv-Rep all reduce model computational complexity. Among them, PConv-Rep performs the best, achieving an mAP50 of 83.7% and increasing FPS to 82.6, while reducing FLOPs and Params to 44.1 G and 14.25 M, respectively. Compared with Faster-Block and DualConv, PConv-Rep not only has fewer parameters and lower computational cost, but also achieves higher detection accuracy. This indicates that its partial convolution and re-parameterized structure can effectively reduce redundant computation and enhance the model’s ability to extract local textures, edge details, and irregular morphological features of ore.

In the comparison of feature fusion modules, HSFPN, BiFPN, and CFPT all improve the multi-scale feature representation capability of the model. HSFPN achieves an mAP50 of 83.4%, while BiFPN performs well in terms of FPS, reaching 82.1. CFPT obtains the highest mAP50 of 84.2%, and its Recall also increases to 86.5%. This indicates that, through cross-layer channel attention and cross-layer spatial attention, CFPT can more fully promote information interaction among features at different scales, enabling the model to more accurately recognize graphite ores of different grades under complex backgrounds. Although CFPT has slightly higher computational cost than BiFPN, its accuracy improvement is more pronounced, making it more suitable for the graphite ore grade recognition task in this study, where detection accuracy and robustness are the primary considerations.

Overall, the three modules optimize the model from the perspectives of bounding box regression, backbone feature extraction, and multi-scale feature fusion, respectively. Wise-Focaler-MPDIoU improves localization accuracy, PConv-Rep reduces computational redundancy and enhances detailed feature extraction, and CFPT strengthens cross-scale semantic fusion. Although the modules function in different ways, they form a complementary relationship in the final model, providing experimental evidence for the overall improvement of OGI-RT-DETR in detection accuracy, inference speed, and lightweight deployment.

### 4.3. Comparative Experiments

To systematically verify the overall performance of OGI-RT-DETR in graphite ore grade detection, it was compared with several mainstream object detection models, including SSD, Faster R-CNN, YOLOv5, YOLOv8, YOLOv10, YOLOv11, and lightweight variants of RT-DETR. All models were trained and tested under the same experimental environment and dataset partition, with unified training configurations maintained to ensure the fairness and comparability of the results. The experimental results are shown in Table 5.

In terms of detection accuracy, OGI-RT-DETR achieves the best results in P, R, and mAP50, reaching 85.6%, 87.6%, and 86.1%, respectively. Compared with the baseline RT-DETR-r18, OGI-RT-DETR improves mAP50 by 3.7 percentage points, indicating that the proposed improvements, including PConv-Rep, CFPT, and Wise-Focaler-MPDIoU, effectively enhance the model’s ability to represent fine-grained features of graphite ore. Compared with the YOLO-series models, OGI-RT-DETR also shows a clear advantage in mAP50, outperforming YOLOv5s, YOLOv8s, YOLOv10s, and YOLOv11n by 6.0, 3.6, 3.3, and 4.8 percentage points, respectively. This demonstrates its greater stability in grade category discrimination and recognition under complex backgrounds.

In terms of inference efficiency and model complexity, lightweight models such as YOLOv5n, YOLOv8n, YOLOv10n, and YOLOv11n have lower FLOPs and Params, and therefore achieve relatively higher FPS. However, their detection accuracy is markedly lower than that of OGI-RT-DETR. Although Faster R-CNN has strong feature modeling capability, its FLOPs reach 371.6 G, Params are 137.2 M, and FPS is only 42.5, making it difficult to meet the real-time detection requirements of industrial sites. In contrast, OGI-RT-DETR achieves the highest mAP50 while maintaining an inference speed of 84.5 FPS and controlling FLOPs and Params at 39.7 G and 12.39 M, respectively. This indicates that the proposed model achieves a favorable balance among accuracy, speed, and model size.

In addition to the general-purpose object detection models listed above, OGI-RT-DETR is also related to recent mineral recognition, ore classification, and transformer-based geological image analysis methods. Previous graphite ore detection studies based on Faster R-CNN and YOLO variants have shown that deep learning can effectively improve ore grade recognition; however, two-stage detectors usually incur higher computational cost, while YOLO-based methods mainly emphasize inference speed and often rely on CNN-style feature aggregation. By contrast, OGI-RT-DETR retains the end-to-end detection paradigm of RT-DETR and further optimizes backbone redundancy, cross-scale feature fusion, and bounding box regression for the fine-grained textures and irregular boundaries of graphite ore. In addition, many recent mineral recognition and ore classification studies focus on image-level classification rather than object-level localization, which limits their direct applicability to ore sorting scenarios. Transformer-based geological image analysis methods provide strong global dependency modeling ability, but they are usually designed for lithology recognition or remote-sensing interpretation rather than real-time visible-light ore detection. Therefore, the proposed framework is positioned as a task-specific detection model that jointly considers localization accuracy, inference speed, and lightweight deployment for graphite ore sorting.

**Figure 9:**
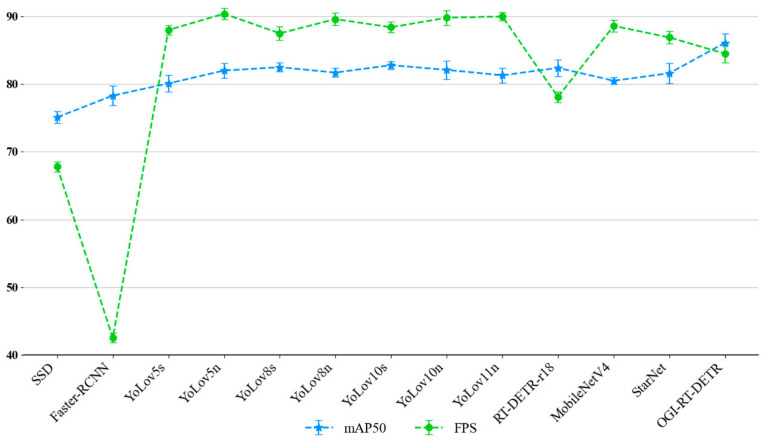
Line-and-point comparison of the detection performance of OGI-RT-DETR and other models.

As shown in Figure 9, different object detection models exhibit obvious differences in terms of mAP50 and FPS. The error bars in the figure represent the standard deviation ranges obtained from three independent experiments and are used to measure the performance fluctuations of different models in repeated trials. It can be observed that OGI-RT-DETR maintains small fluctuations in both mAP50 and FPS, indicating that its performance improvement has good statistical stability and is not caused by random variation in a single experiment.

Among traditional detection models, Faster R-CNN has certain feature modeling capability, but its FPS is significantly low, making it difficult to meet the real-time requirements of online graphite ore detection. SSD achieves a higher inference speed than Faster R-CNN, but its mAP50 remains relatively low, indicating limited recognition capability for complex ore textures and fine-grained grade differences. The YOLO-series models generally show high inference speed, especially lightweight models such as YOLOv5n, YOLOv8n, YOLOv10n, and YOLOv11n, whose FPS values remain in a high range; however, their corresponding detection accuracy does not reach the optimal level. Larger models such as YOLOv8s and YOLOv10s improve mAP50 to some extent, but they still lag behind the proposed model in terms of accuracy stability and grade discrimination capability. RT-DETR-r18 outperforms some traditional and lightweight models in detection accuracy, but its FPS is relatively low, indicating that the original structure still has room for optimization in real-time deployment.

In contrast, OGI-RT-DETR achieves the highest mAP50 in the figure while maintaining a high FPS, with a relatively small error range, demonstrating its advantages in both accuracy improvement and inference stability. Overall, OGI-RT-DETR does not simply pursue the highest speed; instead, it significantly improves detection accuracy while ensuring real-time performance. This indicates that the proposed improvement strategies can effectively enhance the model’s representation capability for key graphite ore features and achieve a better balance between detection accuracy and inference efficiency.

Overall, OGI-RT-DETR does not simply pursue the highest FPS or the smallest number of parameters. Instead, it significantly improves graphite ore grade recognition accuracy while maintaining real-time detection capability. For industrial tasks such as ore sorting, which require both accuracy and real-time performance, the proposed model has stronger practical value and is more suitable for deployment on resource-constrained edge detection devices.

### 4.4. Visualization Experiments

To further intuitively verify the actual detection performance of OGI-RT-DETR in graphite ore grade recognition, this study selected YOLOv11n, the baseline RT-DETR-r18 model, and the improved OGI-RT-DETR model for visual comparison experiments. The experimental samples were randomly selected from the test set and covered three categories of graphite ore, namely low-grade, medium-grade, and high-grade ore. The samples also included different appearance morphologies, illumination conditions, and surface texture characteristics to approximate real industrial sorting scenarios as closely as possible. By comparing the detection box positions, category predictions, and confidence scores of different models, the recognition differences among the models under complex backgrounds can be more clearly observed.

**Figure 10:**
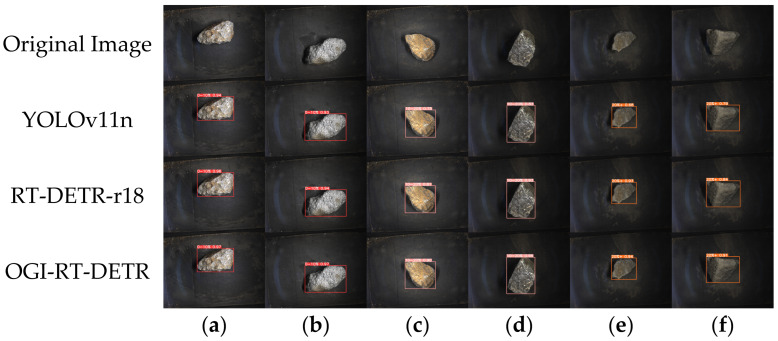
Visual comparison of detection results produced by different models: (**a**,**b**) show low-grade samples, (**c**,**d**) show medium-grade samples, and (**e**,**f**) show high-grade samples.

As shown in Figure 10, different colors are used to distinguish detection results for different grade categories. Red detection boxes represent low-grade graphite ore, pink detection boxes represent medium-grade graphite ore, and orange detection boxes represent high-grade graphite ore. The confidence score beside each detection box indicates the reliability of the current prediction, with a higher value suggesting that the model is more certain about the target category. For graphite ore grade detection, the confidence score not only reflects the classification capability of the model, but also, to some extent, represents the model’s comprehensive understanding of ore boundaries, texture, and color features.

The visualization results show that YOLOv11n and RT-DETR-r18 can complete basic detection for most samples, but they still have certain limitations in some complex scenarios. For example, when strong reflections, dark shadows, or oxidized regions appear on the ore surface, the models are easily affected by local color variations, resulting in less accurate fitting of detection boxes or lower confidence scores. In particular, when the appearance differences between high-grade and medium-grade ores are weak, the baseline model still has limited discriminative capability for fine-grained textures and edge morphologies, which may lead to unstable category prediction.

In contrast, OGI-RT-DETR exhibits better detection stability and category discrimination capability in the visualization results. Its predicted boxes fit the ore target boundaries more closely, and its confidence scores are generally higher than those of YOLOv11n and RT-DETR-r18, indicating that the improved model can more fully extract key features such as ore surface texture, edge contours, and local reflections. Benefiting from the improved backbone feature extraction efficiency provided by PConv-Rep, the enhanced multi-scale feature fusion capability of CFPT, and the optimized bounding box regression process enabled by Wise-Focaler-MPDIoU, OGI-RT-DETR maintains high detection accuracy when facing background interference, irregular ore morphology, and subtle grade-related differences.

The visualization experiments further demonstrate the advantages of OGI-RT-DETR in practical graphite ore grade recognition scenarios. The model can not only accurately identify ores of different grades but also maintain high confidence and favorable bounding box localization under complex backgrounds. Compared with YOLOv11n and RT-DETR-r18, OGI-RT-DETR shows stronger anti-interference capability and more stable fine-grained feature discrimination, demonstrating its application potential in industrial ore sorting and online detection tasks.

In recent years, explainable artificial intelligence (XAI) techniques have been widely applied in industrial vision tasks to reveal the internal feature response mechanisms of deep networks. Among them, Gradient-weighted Class Activation Mapping (Grad-CAM) [59] can generate class response heatmaps using gradient information, thereby intuitively showing the regions that the model focuses on during prediction. Compared with traditional detection result analysis, Grad-CAM can not only indicate whether the model completes target recognition, but also further reveal why the model is able to recognize the target. Therefore, it has important interpretability value in complex industrial scenarios.

To more intuitively verify the effectiveness of the proposed OGI-RT-DETR in graphite ore grade detection, this study uses Grad-CAM to visualize and analyze heatmaps of the models before and after improvement. Grad-CAM generates attention response regions by calculating the weighted relationship between feature-layer gradients and feature maps. Regions closer to red indicate greater contribution to the model prediction, whereas blue regions indicate lower model attention.

**Figure 11:**
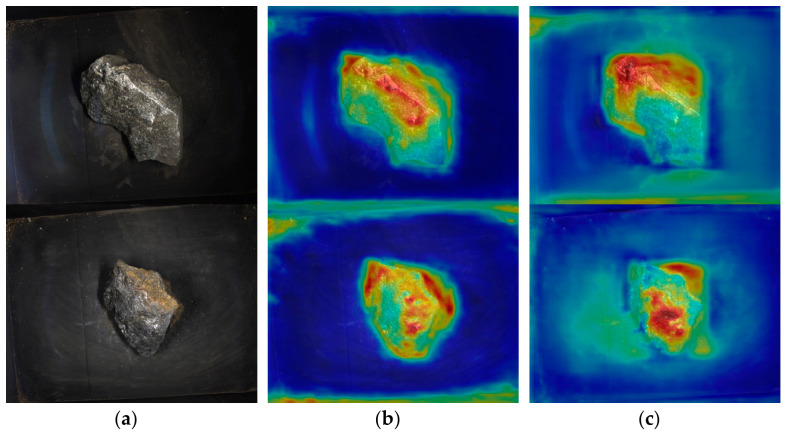
Comparison of heatmap results: (**a**) Original image; (**b**) RT-DETR; (**c**) OGI-RT-DETR.

Figure 11 presents the heatmap visualization results of the models before and after improvement during graphite ore grade detection, allowing comparison of their attention differences in key regions. As shown in Figure 11, the response of the original RT-DETR in the target region is relatively scattered. Although it can attend to the main ore body, the high-response regions are not sufficiently concentrated on the core texture, edge contours, and grade-related feature regions of the ore, indicating that its fine-grained feature focusing capability remains insufficient. In contrast, the improved OGI-RT-DETR produces more concentrated heatmap responses. Its high-response regions mainly cover the ore body and discriminative local regions, enabling it to more prominently capture effective features related to grade recognition. This result indicates that OGI-RT-DETR has better attention selection capability and key feature representation capability in complex scenarios.

Relying only on Grad-CAM heatmaps for analysis is qualitative and makes it difficult to objectively measure differences in the attention-focusing capability of different models. Therefore, based on the visualization analysis, this study further introduces four quantitative metrics to objectively evaluate model attention distributions, including Attention-IoU, foreground attention ratio (FAR), background suppression index (BSI), and energy concentration score (ECS). Among them, FAR is used to measure the proportion of model attention within the foreground target region; BSI reflects the model’s ability to suppress irrelevant responses in the background region; and Attention-IoU and ECS are used to evaluate the consistency between heatmaps and ground-truth target regions in terms of spatial overlap and energy concentration, respectively.

To ensure the reproducibility of the quantitative heatmap analysis, the four attention distribution evaluation metrics are uniformly defined in this study. Let A(x,y) denote the normalized attention response map output by Grad-CAM, where A(x,y)∈[0, 1] represents the attention response intensity of pixel (x,y) in the image. Let G(x,y)∈[0, 1] denote the binary mask corresponding to the ground-truth annotation region. When $G\left(x,y\right)$ = 1, the pixel belongs to the foreground target region; when $G\left(x,y\right)$ = 0, the pixel belongs to the background region. The pixel domain of the whole image is denoted as Ω.

First, the normalized attention map A(x,y) is thresholded to convert continuous response values into a binary form, yielding the binary attention map $A_{b}(x,y)$:

(31) $$A_{b}(x,y)=\left\{\begin{array}{ll} 1, & A(x,y)\geq \tau ,\\ 0, & A(x,y)<\tau , \end{array}\right.,$$

where τ denotes the threshold parameter, which is uniformly set to 0.5 in this study.

Attention-IoU and ECS measure the consistency between the attention heatmap and the ground-truth target region from the perspectives of spatial overlap and response energy concentration, respectively. They are calculated as follows:

(32) $$Attention-IoU=\frac{\sum_{(x,y)\in \Omega }A_{b}(x,y)\cdot G(x,y)}{\sum_{(x,y)\in \Omega }\left[A_{b}(x,y)+G(x,y)-A_{b}(x,y)\cdot G(x,y)\right]},$$

(33) $$ECS=\frac{\sum_{(x,y)\in \Omega }A(x,y{)}^{2}\cdot G(x,y)}{\sum_{(x,y)\in \Omega }A(x,y{)}^{2}}.$$

FAR is used to describe the proportion of model attention falling within the foreground target region, and is calculated as follows:

(34) $$FAR=\frac{\sum_{(x,y)\in \Omega }A(x,y)\cdot G(x,y)}{\sum_{(x,y)\in \Omega }A(x,y)},$$

BSI is used to measure the suppression degree of model responses in irrelevant background regions, and is defined as:

(35) $$BSI=1-\frac{\sum_{(x,y)\in \Omega }A(x,y)\cdot (1-G(x,y))}{\sum_{(x,y)\in \Omega }A(x,y)}.$$

The above four metrics are all calculated at the pixel level based on the normalized attention map and the ground-truth annotation mask, and the entire process does not involve any additional learnable parameters. In general, higher metric values indicate better matching between model attention and the true target region.

**Figure 12:**
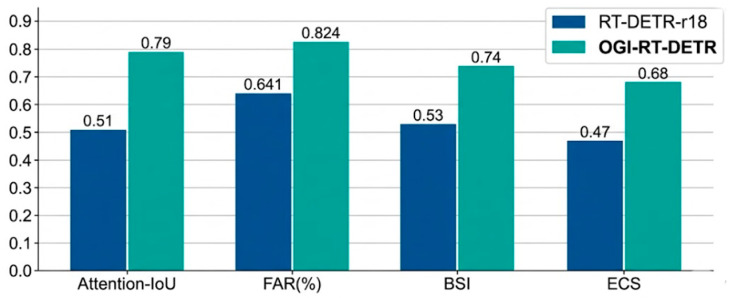
Quantitative evaluation of attention distribution metrics for RT-DETR and the proposed OGI-RT-DETR.

Figure 12 presents the quantitative comparison results of RT-DETR-r18 and OGI-RT-DETR across the four attention metrics. It can be seen that OGI-RT-DETR clearly outperforms the baseline model in all metrics. Specifically, FAR increases from 0.641 to 0.824, corresponding to an improvement of 28.5%, indicating that the improved model can concentrate more attention on the ore body region. BSI increases from 0.53 to 0.74, representing an improvement of approximately 39.6%, which indicates that the model’s ability to suppress complex background textures and noisy regions is significantly enhanced. ECS increases from 0.47 to 0.68, with an improvement of 44.7%, suggesting that the high-response regions generated by the model are more concentrated. Attention-IoU increases from 0.51 to 0.79, corresponding to an improvement of 54.9%, demonstrating higher consistency and spatial overlap between the heatmaps and the true ore regions.

By integrating the qualitative and quantitative results, it can be found that OGI-RT-DETR can not only localize graphite ore regions more accurately, but also effectively reduce the risk of false detections caused by background interference. This indicates that the proposed optimization strategies enhance global information interaction while improving the model’s discriminative capability for key textures and edge structures, thereby providing more reliable feature representation and regional attention mechanisms for graphite ore grade detection in complex industrial scenarios.

### 4.5. Generalization Experiment

To further verify the generalization capability of OGI-RT-DETR in cross-dataset scenarios, this study evaluated the model on a public rock image dataset [60]. This dataset contains 1180 images covering three common rock categories: limestone, mudstone, and sandstone. Compared with the self-built graphite ore dataset used in this study, the public dataset differs in target categories, texture morphology, color distribution, and imaging conditions. Therefore, it can be used to examine the adaptability of the model in cross-domain mineral image recognition tasks.

As shown in Table 6, OGI-RT-DETR still achieves the best detection performance on the public rock dataset, with P, R, and mAP50 values of 72.1%, 73.4%, and 73.6%, respectively. Compared with YOLOv11n, OGI-RT-DETR improves mAP50 by 3.8 percentage points. Compared with the baseline RT-DETR-r18, mAP50 increases by 2.3 percentage points, while FPS improves from 78.5 to 83.9. These results indicate that the improved model can maintain good detection accuracy and inference efficiency in cross-dataset testing. Although YOLOv11n has lower FLOPs and fewer parameters and achieves a higher FPS, its mAP50 is only 69.8%, suggesting that an overly lightweight structure may have insufficient feature representation capability when recognizing complex rock textures.

In terms of model complexity, OGI-RT-DETR has FLOPs of 39.7 G and 12.39 M parameters, which are substantially lower than the 58.2 G FLOPs and 20.04 M parameters of RT-DETR-r18, corresponding to reductions of approximately 31.8% and 38.2%, respectively. This indicates that OGI-RT-DETR maintains good lightweight advantages while improving generalization accuracy. The results demonstrate that the compression of redundant computation by PConv-Rep, the enhancement of cross-scale feature interaction by CFPT, and the optimization of bounding box regression by Wise-Focaler-MPDIoU are not only effective for graphite ore grade recognition, but can also be transferred to other rock image detection tasks.

**Figure 13:**
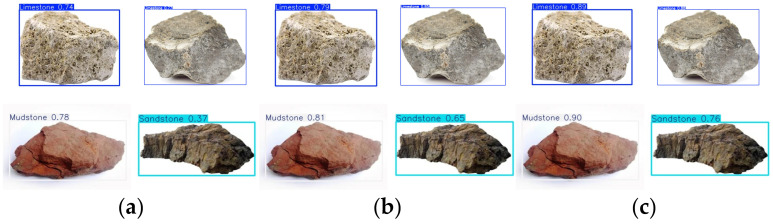
Visual comparison of detection results on the public rock image dataset: (**a**) YOLOv11n; (**b**) RT-DETR; (**c**) OGI-RT-DETR.

Overall, the performance of OGI-RT-DETR on the public rock image dataset demonstrates its good cross-domain generalization capability. When facing targets such as limestone, mudstone, and sandstone, which differ markedly in appearance from graphite ore, the model still maintains high detection accuracy and stable inference speed. This indicates that the texture, edge, and morphological features learned by the model have a certain degree of generality. In addition, the visualization results in Figure 13 further verify this finding. OGI-RT-DETR can accurately localize target regions across different rock categories and provide stable category prediction results, demonstrating strong feature transfer capability and cross-domain mineral detection potential. These results also indicate that OGI-RT-DETR is not only suitable for graphite ore grade detection but also has potential application value in broader mineral image recognition and industrial edge detection scenarios.

### 4.6. Limitations

Although the proposed OGI-RT-DETR achieves favorable detection accuracy, inference speed, and lightweight performance in graphite ore grade recognition, the current study still has several aspects that require further improvement from the perspective of practical industrial application. Clarifying these limitations helps provide a more objective understanding of the applicability of this work and offers directions for future research.

- The dataset scale, geological variability, and operational coverage remain somewhat limited. Although the dataset was collected from real mining environments, the original images were mainly obtained from a specific mining area. Therefore, the represented ore composition, oxidation degree, associated minerals, geological background, and imaging conditions may not fully cover the diversity encountered in other mines or beneficiation sites. This limitation may affect the model’s generalization performance when applied to graphite ores with different mineralogical characteristics, surface textures, reflectance properties, or production conditions. Further validation using multi-source datasets from different deposits and industrial operating environments is therefore necessary.
- Edge device deployment validation is not yet sufficient. This study demonstrates the lightweight advantages of OGI-RT-DETR using metrics such as FLOPs, Params, and FPS, indicating that it has certain potential for edge deployment. However, the current experiments were mainly conducted on a workstation platform, and hardware deployment tests have not yet been performed on NVIDIA Jetson, industrial edge computing devices, or actual sorting systems. Therefore, the inference latency, power consumption, and long-term operational stability of the model on real devices still need further evaluation.
- The input data modality is relatively single. The current model mainly relies on visible-light RGB images for graphite ore grade recognition, which has the advantages of convenient acquisition, low cost, and relatively simple deployment. However, for ores of different grades with highly similar appearances, relying only on color, texture, and morphology information may still be insufficient. Hyperspectral, near-infrared, or XRF sensing may provide additional mineral composition information, but their introduction would substantially change the current visible-light-based sensing framework. Such multimodal extensions would require redesigning the acquisition system, data representation, feature fusion strategy, and possibly the model architecture. Therefore, they should be regarded as a separate future research direction rather than a direct extension of the present lightweight RGB image-based approach.
- Validation in dynamic conveyor-belt sorting scenarios still needs to be strengthened. Although the graphite ore images used in this study were collected from real mining environments, the samples shown in Figure 5 are mainly isolated and static ore images. They are useful for analyzing grade-related visual features, such as color, texture, edge contours, and surface morphology, but cannot fully represent conveyor-belt conditions involving dense ore distribution, overlap, occlusion, motion blur, background changes, and dynamic illumination. In addition, the current data augmentation strategies mainly simulate illumination variation, background interference, posture changes, scale variation, and boundary ambiguity, but cannot fully reproduce continuous conveyor-belt operation. Therefore, future work will collect conveyor-belt images and video data, introduce more realistic augmentation strategies, and evaluate the model in continuous production environments.

Future research will focus on expanding conveyor-belt and multi-source graphite ore datasets and deploying the model on real edge devices to further improve the generalization capability and practical application value of OGI-RT-DETR in complex industrial scenarios. In addition, multimodal sensing technologies, such as hyperspectral, near-infrared, and XRF sensing, may be explored as a separate extension in future studies, with corresponding redesign of the sensing system and model framework.

## 5. Conclusions

To address the complex detection procedures and insufficient real-time performance of traditional graphite ore grade detection methods, as well as the difficulty of existing object detection models in balancing accuracy, speed, and lightweight deployment, this study proposed OGI-RT-DETR, a graphite ore grade recognition model based on an improved RT-DETR. Oriented toward graphite ore detection requirements in complex industrial scenarios, the proposed model improves RT-DETR-r18 from three aspects: backbone feature extraction, neck feature fusion, and bounding box regression optimization.

First, the PConv-Rep module was introduced into the backbone network. Partial convolution was used to reduce redundant computation, and structural re-parameterization was incorporated to enhance the model’s feature extraction capability for ore edges, local textures, and irregular morphologies. Second, the CFPT module was adopted to replace the original CCFM structure. Cross-layer channel attention and cross-layer spatial attention were used to strengthen information interaction among features at different scales, thereby improving the model’s representation capability for complex backgrounds and multi-scale ore targets. Finally, the Wise-Focaler-MPDIoU loss function was introduced to optimize the bounding box regression process, enabling the model to focus more effectively on high-quality samples and geometric information of ore boundaries, thereby improving localization accuracy and training stability.

Experiments were conducted on a self-built graphite ore image dataset, and data augmentation was used to simulate complex industrial acquisition environments. The experimental results showed that OGI-RT-DETR achieved Precision, Recall, and mAP50 values of 85.6%, 87.6%, and 86.1%, respectively. Compared with the baseline RT-DETR-r18, mAP50 increased by 3.7 percentage points, FPS improved by approximately 8.2%, and FLOPs and Params decreased by approximately 31.8% and 38.2%, respectively. Ablation experiments, comparative experiments, confusion matrices, and visualization results further demonstrated that the improved modules can collaboratively enhance the model’s ability to recognize fine-grained features of graphite ore and effectively alleviate confusion among ores of different grades.

Overall, OGI-RT-DETR effectively reduces model complexity while improving detection accuracy, and also shows favorable real-time and lightweight performance. This method provides an efficient and feasible vision-based detection solution for automatic graphite ore grade detection, and offers a reference for the application of object detection models in intelligent mineral sorting and industrial edge deployment. However, the current study still has several limitations. The dataset size remains relatively limited; the images were mainly collected from a single mining source, and the model has not yet been fully validated under real industrial operating conditions, such as continuous conveyor-belt sorting, dense ore distribution, motion blur, and dynamic illumination. Future research will further expand multi-source and conveyor-belt graphite ore datasets and verify the deployment performance and robustness of the model on real edge devices and in continuous production scenarios. In addition, multimodal sensing technologies, such as hyperspectral, near-infrared, and XRF sensing, may be explored as a separate future research direction, but their introduction would require a corresponding redesign of the sensing system, data representation, and model framework.

## Figures and Tables

**Table 1:** Details of the dataset partitioning.

	Train Set	Valid Set	Test Set	Total
Total	2951	843	422	4216
0–10%	1184	346	174	1704
10–20%	1176	329	164	1669
20%+	591	168	84	843

**Table 2:** Hardware and software configuration of the experimental environment.

Name	Parameter
System	Windows 10 (64 bit)
CPU	Intel64 Family 6 Model 85 Stepping 7
Memory	31 GB
GPU	NVIDIA GeForce RTX 3080 Ti
Video Memory	11 GB
Programming Software	Python 3.10.15
Deep Learning Framework	PyTorch 2.5.1
GPU Acceleration Library	CUDA11.8

**Table 4:** Ablation study results for individual modules.

Model	P (%)	R (%)	mAP50 (%)	FPS	FLOPs (G)	Params (M)
Wise-IoU	83.1	85.5	82.8	78.2	58.2	20.04
Focaler-IoU	83.4	85.7	83.0	78.0	58.4	20.11
MPDIoU	83.5	85.8	83.1	77.9	58.6	20.15
Wise-Focaler-MPDIoU	83.9	85.9	83.4	78.3	58.3	20.08
Faster-Block	83.6	85.4	82.9	80.4	50.7	16.99
DualConv	83.9	85.8	83.2	81.5	45.6	16.08
PConv-Rep	84.1	86.2	83.7	82.6	44.1	14.25
HSFPN	83.8	86.0	83.4	79.5	54.4	18.22
BiFPN	83.5	86.3	83.6	82.1	47.5	15.61
CFPT	83.7	86.5	84.2	80.8	53.6	18.24

**Table 5:** Comparative experimental results.

Methods	P (%)	R (%)	mAP50 (%)	FPS	FLOPs (G)	Params (M)
SSD [54]	69.5	80.2	75.1	67.8	53.2	24.5
Faster-RCNN	76.8	82.1	78.3	42.5	371.6	137.2
YoLov5s [55]	75.2	79.0	80.1	88.0	23.3	10.13
YoLov5n [55]	77.4	79.5	82.0	90.4	7.5	2.78
YoLov8s [56]	80.1	81.3	82.5	87.5	27.9	12.03
YoLov8n [56]	76.5	78.8	81.7	89.6	8.6	3.16
YoLov10s [57]	80.8	82.1	82.8	88.4	24.2	8.03
YoLov10n [57]	76.9	79.3	82.1	89.8	8.4	2.93
YoLov11n [58]	76.0	80.1	81.3	90.0	7.8	2.81
RT-DETR-r18	83.2	85.7	82.4	78.1	58.2	20.05
RT-DETR-MobileNetV4	81.3	84.2	80.5	88.6	40.6	11.52
RT-DETR-StarNet	82.7	84.8	81.6	86.9	32.9	12.20
OGI-RT-DETR	85.6	87.6	86.1	84.5	39.7	12.39

**Table 6:** Comparative performance of different models on the public rock image dataset.

Model	P (%)	R (%)	mAP50 (%)	FPS	FLOPs (G)	Params (M)
YoLov11n	66.2	68.5	69.8	90.4	7.8	2.82
RT-DETR-r18	69.7	70.8	71.3	78.5	58.2	20.04
OGI-RT-DETR	72.1	73.4	73.6	83.9	39.7	12.39

## Data Availability

Data supporting the results of this study are available from the corresponding author upon request.
